# Serum hepcidin and erythropoietin in relation to anemia in non-diabetic chronic kidney disease: a cross-sectional study

**DOI:** 10.1038/s41598-026-68785-z

**Published:** 2026-09-12

**Authors:** Mostafa Gamal, A. M. Abdel-Rahman, Ahmed Abd Elwahab, Huda Refaie, Sawsan M. El-Halawani, Wael l. Mortada, Mohamed Sobh

**Affiliations:** 1 Clinical Nephrology and Kidney Transplantation Department, Urology and Nephrology Center, Mansoura University, Mansoura, 35516 Egypt; 2 Mansoura Nephrology and Dialysis Unit, Mansoura University, Mansoura, 35516 Egypt; 3 Radiology Department, Urology and Nephrology Center, Mansoura University, Mansoura, 35516 Egypt; 4 Biotechnology Department, Urology and Nephrology Center, Mansoura University, Mansoura, 35516 Egypt; 5 Clinical Chemistry Lab, Urology and Nephrology Center, Mansoura University, Mansoura, 35516 Egypt

**Keywords:** Diseases, Medical research, Nephrology

## Abstract

Chronic kidney disease (CKD) is a global health problem that disrupts iron homeostasis and leads to anemia. The complex interactions among hepcidin, erythropoietin, and anemia play important roles in CKD management. Increased hepcidin levels in CKD patients disrupt iron bioavailability for erythropoiesis, whereas altered erythropoietin production affects red blood cell synthesis. This study explored the interaction between these factors in a cohort of 100 nondiabetic CKD patients. A cross-sectional study was conducted on 100 nondiabetic CKD patients at the Urology and Nephrology Center, Mansoura University, Egypt. Crucially, all included patients were completely naive to prior iron supplementation and erythropoiesis-stimulating agents (ESAs), eliminating these major therapeutic confounders. Hemoglobin levels were determined to create two groups: anemia (Hb < 12 g/dl) and non-anemia (Hb ≥ 12 g/dl). Patients were categorized by CKD stage (KDIGO eGFR categories); patients with active systemic bacterial infection were excluded. Multivariable linear regression was used to examine independent associations between hemoglobin and serum hepcidin after adjusting for age, sex, eGFR, TSAT, ferritin, iPTH, serum hepcidin, and erythropoietin (EPO). Of 100 enrolled, 6 were lost to follow-up; 94 were analysed (mean age 53.6 ± 11.2 years; 71.3% male). Hepcidin and EPO were available in 81 of 94 (86%). Serum ferritin levels were comparable between the anemic and nonanemic groups (median 98.5 [31.3–150.65] ng/ml vs. 88.26 [53.3–145.38] ng/ml; *p* = 0.753). Hepcidin levels were slightly higher in anemic patients (median 226.75 [202–268.38] ng/ml) than in nonanemic patients (196 [170.25–247.25] ng/ml), but the difference was not statistically significant (*p* = 0.077). Transferrin saturation (TSAT) was lower in the anemia group (median 23.4% [19.35–31.76]) compared to the control group (26.37% [21.45–31.52]), yet this difference also did not reach statistical significance (*p* = 0.282). Hepcidin levels remained statistically comparable across the varying stages of CKD and showed a significant negative correlation with hemoglobin (ρ = -0.277, *p* = 0.012), whereas TSAT demonstrated a modest positive correlation (ρ = 0.188, *p* = 0.071). In the adjusted regression model, TSAT was independently associated with hemoglobin (β = 0.047, 95% CI 0.012–0.082, *P* = 0.009), while hepcidin and erythropoietin were not. Transferrin saturation is independently associated with hemoglobin levels in non-diabetic chronic kidney disease, whereas serum hepcidin and erythropoietin are not. These findings indicate that functional iron bioavailability, rather than circulating hepcidin or erythropoietin, is the primary correlate of hemoglobin variance in this cohort.

**Supplementary Information:**

The online version contains supplementary material available at https://doi.org/10.1038/s41598-026-68785-z.

## Introduction

Chronic kidney disease (CKD) is a major global health burden, affecting approximately > 10% of the adult population worldwide, amounting to > 800 million individuals and representing a leading cause of morbidity and mortality^1^. Non-diabetic CKD accounts for approximately 60–70% of all CKD cases globally^2^. Anemia is one of the most common complications of CKD and is strongly associated with reduced quality of life, increased hospitalization, and adverse cardiovascular outcomes. Among the various mechanisms contributing to anemia in CKD, disturbances in iron homeostasis and impaired erythropoietin (EPO) production play central roles^3^.

Reduced renal EPO synthesis is a well-established primary driver of anemia in CKD, leading to inadequate stimulation of erythropoiesis as kidney function declines. However, anemia in CKD is multifactorial and is additionally influenced by iron deficiency, inflammation, nutritional abnormalities, and impaired iron mobilization^4^. Hepcidin, a hepatic peptide hormone, is a key regulator of systemic iron metabolism. It limits dietary iron absorption and iron release from macrophages by binding to ferroportin and inducing its degradation. Elevated hepcidin levels, commonly observed in CKD due to reduced renal clearance and increased inflammation, reduce iron availability for erythropoiesis and contribute to functional iron deficiency^5^.

Inflammation further amplifies anemia severity by stimulating hepcidin production through IL-6–mediated pathways and by impairing iron utilization^6^. Nutritional deficiencies (iron, B12, folate) and factors such as gastrointestinal blood loss or frequent blood sampling can worsen anemia^7^. Recent research has highlighted the role of novel biomarkers and therapeutic targets in the management of CKD-related anemia. For example, studies have shown that fibroblast growth factor 23 (FGF23) and its coreceptor Klotho are involved in iron metabolism and erythropoiesis regulation. Elevated FGF23 levels are associated with increased hepcidin expression and reduced iron availability, suggesting that FGF23 is a potential therapeutic target for anemia in CKD patients^8^. Additionally, the use of hypoxia-inducible factor prolyl hydroxylase inhibitors (HIF-PHIs) has emerged as a promising treatment for anemia in CKD patients. HIF-PHIs stimulate endogenous EPO production and improve iron metabolism by reducing hepcidin levels. Clinical trials have demonstrated the efficacy of HIF-PHIs in increasing hemoglobin levels and reducing the need for erythropoiesis-stimulating agents (ESAs) in CKD patients^9,10^.

Despite considerable research, the precise interplay between hepcidin, endogenous EPO, and iron indices in non-diabetic CKD remains insufficiently clarified. Many studies have evaluated hepcidin or EPO individually, but fewer have explored their combined effects on anemia severity after accounting for confounders such as inflammation, iron status, and mineral–bone parameters (e.g., PTH). Moreover, most available studies include mixed CKD populations dominated by diabetic nephropathy, which may confound the interpretation of hepcidin–EPO interactions due to diabetes-related inflammation and metabolic alterations. Consequently, data focusing on nondiabetic CKD populations remain limited.

The present study aimed to investigate the complex relationship between hepcidin, erythropoietin, and hemoglobin levels in non-diabetic CKD patients, a subgroup that is less frequently examined despite its clinical importance. By evaluating hepcidin and EPO concurrently and accounting for iron parameters, inflammation, and mineral–bone markers, this study provides a more comprehensive understanding of the multifactorial nature of anemia in CKD. Conducting this research in a well-characterized Egyptian CKD cohort also contributes region-specific data that may enhance the global understanding of anemia pathophysiology in CKD.

## Methods

### Study design and patient selection

This cross-sectional study evaluated the medical records and clinical data of chronic kidney disease (CKD) patients at the Urology and Nephrology Center, Mansoura University, Egypt. The experimental protocols were approved by the Institutional Review Board (IRB) of the Faculty of Medicine, Mansoura University (Approval No. MS.22.04.1973). All methods were performed in accordance with relevant ethical guidelines and regulations.

A total of 100 patients were initially screened. Inclusion criteria required participants to be over 18 years of age with no signs of an acute decline in glomerular filtration rate (GFR) within the previous three months. Furthermore, all included patients were completely naive to prior iron supplementation and erythropoiesis-stimulating agents (ESAs).

Patients were excluded if they had a history of diabetes mellitus (determined via medical records, medication history, or glycated hemoglobin [HbA1c] measurements in the diabetic range), chronic liver disease, or chronic respiratory disorders. Additional exclusion criteria included: active systemic bacterial infection at enrollment (defined by a fever > 38 °C, a positive culture, or antibiotic treatment within 4 weeks prior to sampling); ongoing active cancer; active infection with human immunodeficiency virus (HIV), hepatitis B virus (HBV), hepatitis C virus (HCV), or tuberculosis (TB); primary or secondary glomerulonephritis or nephrotic syndrome with heavy proteinuria (≥ 3.5 g/day); active immunological or collagen vascular disease; or inability/refusal to provide informed consent.

Among the 100 initial eligible candidates, 6 were excluded prior to clinical and laboratory assessments (2 were unavailable for sampling due to traveling abroad, 2 withdrew their consent, and 2 did not attend their scheduled assessment), leaving a final analytical cohort of 94 enrolled patients. The etiology of CKD for each participant was determined using our institutional patient information system, incorporating clinical histories, laboratory investigations, imaging, and available renal biopsy records.

### Clinical definitions and grouping

Participants were categorized by CKD stage according to Kidney Disease: Improving Global Outcomes (KDIGO) estimated GFR (eGFR) categories: stage G2 (60–89 mL/min/1.73 m²), G3a (45–59 mL/min/1.73 m²), G3b (30–44 mL/min/1.73 m²), and G4 (15–29 mL/min/1.73 m²).

Standard WHO and KDIGO clinical practice guidelines define anemia as hemoglobin < 13.0 g/dL for men and < 12.0 g/dL for women^11^, However, for all primary comparative analyses in this study, a uniform operational cutoff of hemoglobin < 12.0 g/dL was intentionally applied across both sexes (Group 1: Anemic [hemoglobin < 12.0 g/dL] vs. Group 2: Non-anemic [hemoglobin ≥ 12.0 g/dL]). This unified threshold was selected based on critical physiological, epidemiological, and diagnostic considerations in non-dialysis CKD cohorts. First, epidemiological data demonstrate that the physiological male-female hemoglobin gap (~ 1.0 g/dL) narrows significantly in CKD populations due to age- and renal-associated androgen decline^12^. Second, in elderly male CKD patients, mild hemoglobin reductions between 12.0 and 12.9 g/dL frequently result from non-renal confounders (e.g., vascular aging, comorbidities, or RAAS inhibitor therapy) rather than primary renal erythropoietic impairment^13^.

### Laboratory measurements and eGFR calculation

Following an overnight fast of at least 8 h, blood samples were drawn from the antecubital vein. Whole blood was collected in commercial lavender-top tubes containing K2EDTA as an anticoagulant. Hemoglobin, hematocrit, mean corpuscular volume (MCV), and mean corpuscular hemoglobin (MCH) were quantified using a Sysmex XN-1000 hematological analyzer. The baseline neutrophil-to-lymphocyte ratio (NLR) was calculated by dividing the absolute neutrophil count by the absolute lymphocyte count from the complete blood count. NLR was utilized as a validated surrogate biomarker of baseline systemic inflammatory status.

For biochemical analyses, blood was collected in standard tubes, allowed to clot, and centrifuged at at 2611 ×g (4000 rpm, Hermle Z 306 centrifuge) for 10 min to separate the serum. An Abbott ARCHITECT c4000 analyzer was used to measure serum creatinine, cholesterol, magnesium, and uric acid, alongside calcium, phosphate, and alkaline phosphatase. A Roche COBAS 6000 was utilized to analyze intact parathyroid hormone (iPTH), ferritin, and transferrin. Additionally, human hepcidin enzyme-linked immunosorbent assay (ELISA) kits (Chongqing Biospes Co., China) were used to measure the total serum hepcidin (Catalog number: BZEK2269, analytical range: 10–160 ng/mL) and erythropoietin (Catalog number: BYEK1485, analytical range: 3–36 mU/mL) levels according to the manufacturer’s instructions. All samples and standards were analyzed under identical experimental conditions. Both hepcidin and erythropoietin assays demonstrated intra-assay and inter-assay coefficients of variation (CVs) of < 10.0% and < 12.0%, respectively, as reported by the manufacturer. Of the 94 analyzed patients, hepcidin and erythropoietin measurements were successfully completed for 81 participants. The remaining 13 missing biomarker values resulted strictly from the fixed capacity of the 96-well commercial ELISA plates, which required 15 wells for calibration standards and quality controls, leaving exactly 81 available wells for patient samples. Because this limitation was purely logistical, the missing data occurred completely at random (MCAR) and was not associated with any clinical or demographic patient characteristics. Samples with hepcidin levels exceeding the upper calibration limit (160 ng/mL) were diluted using the manufacturer’s sample diluent, reanalyzed, and the final concentrations were calculated by applying the appropriate dilution factor.

The eGFR was calculated using the 2009 CKD-EPI (Chronic Kidney Disease Epidemiology Collaboration) creatinine equation^14^. Given the homogenous North African (Egyptian) study population, the race multiplier coefficient was set to 1 (non-Black/other) across all participants to ensure accurate local estimation^15^. The equation used was:

$$ {\mathrm{eGFR}}_{{{\mathrm{cr}}}} = {\mathrm{142}} \times {\mathrm{min}}\left( {{\mathrm{S}}_{{{\mathrm{cr}}}} /\kappa ,{\mathrm{1}}} \right)^{\alpha } \times {\mathrm{max}}\left( {{\mathrm{S}}_{{{\mathrm{cr}}}} /\kappa ,{\mathrm{1}}} \right)^{{ - {\mathrm{1}}.{\mathrm{2}}00}} \times 0.{\mathrm{9938}}^{{{\mathrm{Age}}}} \times {\mathrm{1}}.0{\text{12 }}\left[ {{\text{if female}}} \right]. $$

Where S_cr_ = standardized serum creatinine in mg/dL, κ = 0.7 (females) or 0.9 (males), α = -0.241 (female) or -0.302 (male), min(S_cr_/κ, 1) is the minimum of S_cr_/κ or 1.0, max(S_cr_/κ, 1) is the maximum of S_cr_/κ or 1.0, and Age (years).

### Statistical analysis

#### Sample size calculation

The study sample size was determined a priori to detect a clinically relevant correlation between serum hepcidin and hemoglobin. Utilizing Fisher’s z-transformation approach for a correlation coefficient of *r* = 0.30, with a two-sided significance level of α = 0.05 and statistical power of 80%, the estimated minimum requirement was approximately 85 participants. To account for potential missing data or incomplete laboratory measurements, the target was increased to 90 participants. The final enrollment of 94 patients ensured adequate statistical robustness.

#### Data analysis

Statistical analyses were performed using SPSS software (version 27). Continuous variables were evaluated for normal distribution using the Shapiro–Wilk test and visual distribution plots. Normally distributed continuous variables are presented as means and standard deviations, while non-normally distributed data are expressed as medians with interquartile ranges (25th–75th percentiles). Group comparisons were conducted utilizing the independent samples t-test for normally distributed data and the Mann–Whitney U test for non-normally distributed data. To compare continuous variables across the different CKD stages, one-way ANOVA or the Kruskal-Wallis H test was applied, as appropriate for the data distribution. Categorical variables are presented as frequencies and percentages (%), with differences computed using the Pearson chi-square test or Fisher’s exact test.

#### Multivariable regression and sensitivity analysis

To identify factors independently influencing hemoglobin levels, a multiple linear regression model was constructed using the original variable scales to facilitate clinical interpretation. The covariates included age, gender, eGFR, iPTH, erythropoietin, hepcidin, transferrin saturation, and ferritin. Complete-case analysis was applied; cases with missing values in any regression variable were excluded, resulting in a final regression model based on 80 participants. Furthermore, a sensitivity analysis was conducted by repeating the correlation and regression analyses after excluding an extreme hepcidin outlier (1287.5 ng/mL) to evaluate its influence on the findings. Multicollinearity among the independent predictors in the multivariable linear regression model was formally assessed using the Variance Inflation Factor (VIF), with a conservative VIF threshold of < 3.0 indicating the absence of significant collinearity.

Given the exploratory nature of the study and the multiple bivariate between-group comparisons performed across various parameters, no formal statistical adjustments for multiple testing were applied. Consequently, individual univariable p-values should be interpreted as exploratory, with an inherent risk of Type I error inflation. P-values ≤ 0.05 were considered statistically significant.

## Results

**Fig. 1:**
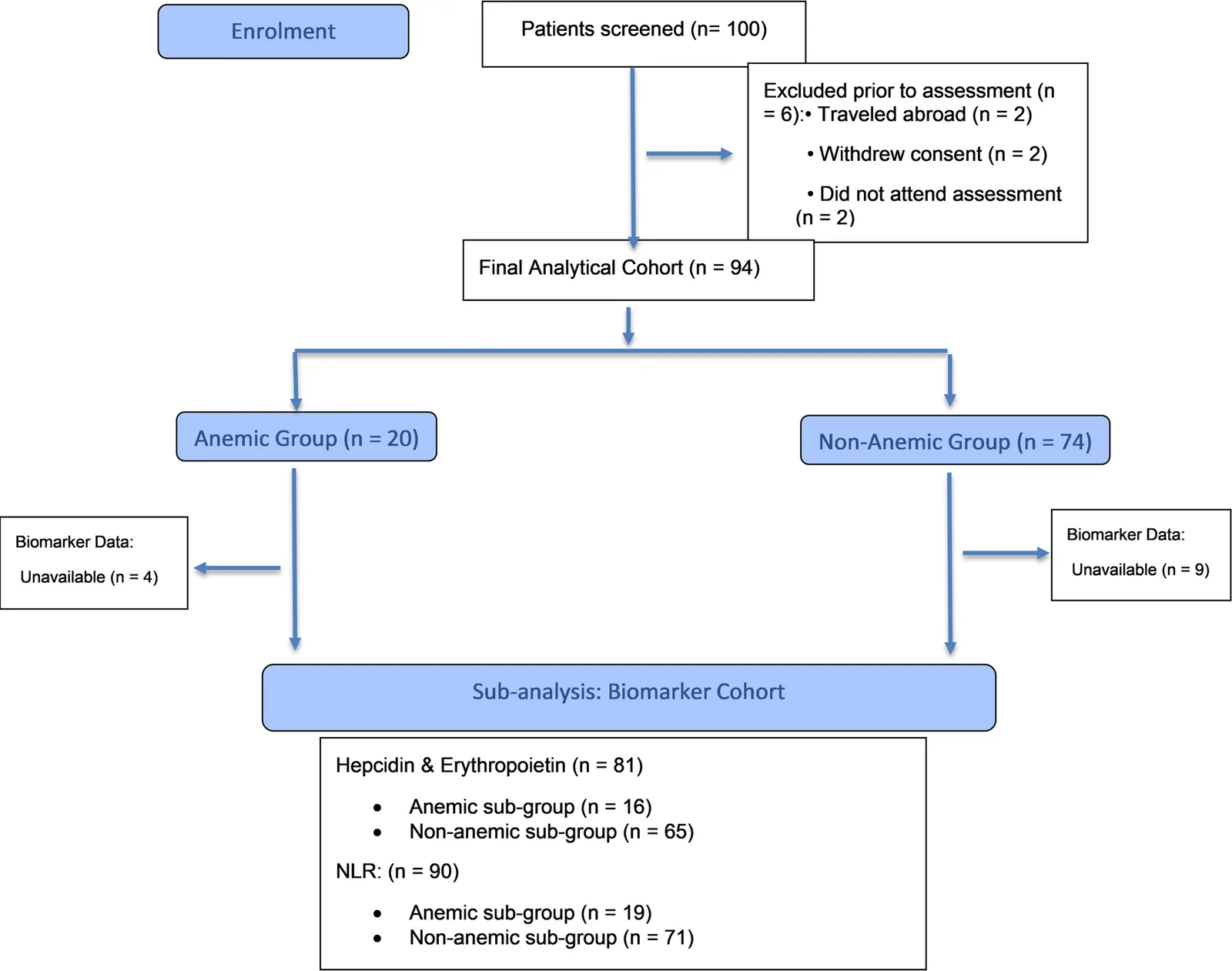
Flowchart of patient screening, clinical grouping, and biomarker data availability.

The screening, exclusion, and final allocation of the study participants are illustrated in the participant flow diagram (Fig. 1). Table 1 presents the demographic and clinical characteristics of the study cohort (*N* = 94). The mean age was 53.6 ± 11.2 years, with a male predominance (71.3%). The most frequent cause of CKD was unknown (50%), followed by stone kidney disease (18.1%) and solitary kidney (13.8%). Hypertension was the most common comorbidity (71.3%), and the average BMI indicated obesity (31.4 ± 5.3 kg/m²). The majority of patients were in CKD stage G3b (51.1%), with fewer in G2/G3a (38.3%) and G4 (10.6%).

**Table 1:** Demographic data.

Variable	*N* = 94
Age (years)	53.57 ± 11.16
Sex (M)	67 (71.3%)
Cause of CKD	
Unknown	47 (50%)
PCK	11 (11.7%)
Solitary kidney	13 (13.8%)
Interstitial nephritis	1 (1.1%)
Stone kidney disease	17 (18.1%)
HTN nephropathy	5 (5.3%)
Comorbidities	
No	8 (8.5%)
HTN	67 (71.3%)
Cardiac	19 (20.2%)
BW (kg)	89.38 ± 14.29
BMI (kg/m^2^)	31.42 ± 5.26
CKD stages	
G2/G3a	36 (38.3%)
G3b	48 (51.1%)
G4	10 (10.6%)

Table 2 compares the biochemical variables between the anemic and nonanemic groups. We found no significant differences in the eGFR, iPTH, alkaline phosphatase, magnesium, calcium, phosphorus, uric acid or lipid profile.

**Table 2:** Biochemical data.

Variable	Anemic (*n* = 20)	Non anemic (*n* = 74)	Mean difference & 95% CI	*p* value
Cr (mg/dl)	1.76 ± 0.4	1.8 ± 0.46	− 0.4 (− 0.26, 0.19)	0.727
eGFR (ml/min)	44 ± 12.36	44 ± 11.78	0.07 (− 5.89, 6.03)	0.982
iPTH (pg/ml)	62.7 (45.43, 101.15)	54.7 (41.03, 80.2)		0.268
Calcium (mg/dl)	9.37 ± 0.41	9.27 ± 0.69	0.1 (− 0.22, 0.42)	0.522
phosphorus (mg/dl)	3.36 ± 0.60	3.18 ± 0.74	0.18 (− 0.18, 0.55)	0.324
ALP (IU/L)	68 (56, 77)	66 (56.75, 81)		0.667
Mg (mg/dl)	1.97 ± 0.18	1.93 ± 0.19	0.04 (− 0.06, 0.14)	0.398
UA (mg/dl)	6.41 ± 1.84	6.9 ± 1.68	− 0.49 (− 1.34, 0.37)	0.264
Cholesterol (mg/dl)	177 (140.75, 232.5)	168 (145.5, 205.5)		0.602
TG (mg/dl)	140 (85.5, 228)	140 (102.5, 184)		0.716
HDL-C (mg/dl)	36 (31.25, 47.25)	38 (32, 44)		0.756
LDL-C (mg/dl)	105.5 (95, 135.75)	110 (90.25, 144)		0.837

Sample sizes vary across variables due to missing laboratory measurements. Sample sizes for biochemical parameters were: phosphorus (*n* = 93), magnesium (*n* = 90), alkaline phosphatase (*n* = 89), total cholesterol (*n* = 94), triglycerides (*n* = 87), HDL-C and LDL-C (*n* = 86), and serum hepcidin and erythropoietin (*n* = 81). All other variables were available for ≥ 93 participants.

Table 3 shows the differences in hematological characteristics between the study groups including CBC, iron profile, hepcidin and erythropoietin levels. Anemic patients presented significantly lower values for hemoglobin and hematocrit (Hgb: 10.92 ± 0.66 vs. 13.86 ± 1.25, *p* < 0.001; Hct: 34.44 ± 2.91 vs. 42.47 ± 3.93, *p* < 0.001). The mean corpuscular volume (MCV) was similar between the groups (*p* = 0.614). Although median iron and transferrin saturation (TSAT) levels are lower in anemic patients, these differences are not statistically significant (*p* = 0.141 and *p* = 0.282, respectively). Ferritin, erythropoietin (EPO), and hepcidin levels also showed no significant differences. Furthermore, baseline systemic inflammation evaluated via the NLR was completely comparable between the anemic and non-anemic groups (median 1.79 [IQR: 1.24–2.58] vs. 1.89 [IQR: 1.47–2.50]; *p* = 0.444), with both groups remaining well within normal physiological limits.

**Table 3:** Hematological characteristics.

Variable	Anemic (*n* = 20)	Non anemic (*n* = 74)	Mean difference & 95% CI	*p* value
Hb (g/dl)	10.92 ± 0.66	13.86 ± 1.25	− 2.94 (− 3.36, − 2.53)	**< 0.001**
Hct (%)	34.44 ± 2.91	42.47 ± 3.93	− 8.03 (− 9.91, − 6.16)	**< 0.001**
MCV (fl.)	84.78 ± 7.31	83.94 ± 6.36	0.84 (− 2.45, 4.12)	0.614
Iron (µg/dl)	62 (48.75, 100)	75 (61, 84.5)		0.141
TSAT (%)	23.4 (19.35, 31.76)	26.37 (21.45, 31.52)		0.282
Ferritin (ng/ml)	98.5 (31.3, 150.65)	88.26 (53.3, 145.38)		0.753
Erythropoietin (mU/ml)	4.69 (3.47, 8.22)	4.7 (3.09, 8.20)		0.644
Hepcidin (ng/ml)	226.75 (202, 268.38)	196 (170.25, 247.25)		0.077
Neutrophil/Lymphocyte (NLR)	1.79 (1.24, 2.58)	1.89 (1.47, 2.5)		0.444

Hepcidin and erythropoietin measurements were available in 81 participants (16 anemic and 65 non-anemic).

For hepcidin: 1 ng/ml = 0.359 nM.

Significant values are in bold.

Table 4 compares hematological, iron regulatory, and mineral-bone parameters across CKD stages G2/G3a, G3b, and G4. While hemoglobin, transferrin saturation (TSAT), erythropoietin (EPO), and hepcidin levels showed no significant differences across stages (*P* = 0.952, 0.785, 0.733 and 0.263, respectively), intact parathyroid hormone (iPTH) levels were markedly elevated in stage G4 patients (median 81.1 pg/mL; *P* = 0.004). In contrast, serum calcium and phosphorus concentrations remained stable across all stages (*P* = 0.161 and 0.568, respectively).

**Table 4:** Distribution of serum erythropoietin (EPO), hepcidin, and intact parathyroid hormone (iPTH) across CKD stages.

	G2/G3a (*n* = 36)	G3b (*n* = 48)	G4 (*n* = 10)	*P* value
Erythropoietin (mU/ml)	4.94 (3.10, 7.05)	5.24 (3.18, 8.85)	3.59 (3.27, 8.60)	0.733
Hepcidin (ng/ml)	210.25 (179.75, 262.38)	219.50 (169.25, 257.75)	191.25 (149.50, 219)	0.263
iPTH (pg/ml)	51.7 (31.7, 65.8)	60.05 (44.1, 95.65)	81.1 (68.13, 111.38)	**0.004**
Hemoglobin (gm/dl)	13.23 ± 1.69	13.29 ± 1.65	13.09 ± 1.63	0.952
TSAT (%)	25 (21.2, 31.1)	25 (18.96, 31.65)	27.25 (22.29, 32.48)	0.785
Calcium (mg/dl)	9.12 ± 0.76	9.38 ± 0.56	9.33 ± 0.43	0.161
Phosphorus (mg/dl)	3.13 ± 0.72	3.24 ± 0.73	3.39 ± 0.65	0.568

Hepcidin and erythropoietin data were available for 34 patients in G2/G3a, 37 in G3b, and 10 in G4.

For hepcidin: 1 ng/ml = 0.359 nM.

Data are presented as mean $\:\pm\:$ standard deviation for normally distributed variables and median (interquartile range) for non-normally distributed variables.

Significant values are in bold.

Table 5 presents the results of the multivariable linear regression analysis. Transferrin saturation (TSAT) was independently associated with hemoglobin levels in the adjusted model (β = 0.047, 95% CI 0.012–0.082, *p* = 0.009). None of the other variables included in the model showed a statistically significant independent association with hemoglobin levels.

**Table 5:** Factors affecting hemoglobin levels in CKD patients: multivariable linear regression (*n* = 80 complete cases).

	Estimate (B)	95% confidence interval for (B)		*p* value	VIF
		Lower bound	Upper bound		
Intercept	13.63395	10.60	16.67	< 0.001	
Age (years)	− 0.00405	− 0.039	0.031	0.819	1.22
Gender	− 0.72040	− 1.51	0.071	0.074	1.11
eGFR (ml/min)	0.00535	− 0.026	0.037	0.735	1.18
iPTH (pg/ml)	− 0.00516	− 0.016	0.005	0.329	1.24
Erythropoietin level (mU/ml)	− 0.01798	− 0.125	0.089	0.739	2.34
Serum hepcidin (ng/ml)	− 0.00122	− 0.005	0.002	0.483	2.2
TSAT (%)	0.04733	0.012	0.082	**0.009**	1.16
Serum ferritin (ng/ml)	0.00064	− 0.004	0.006	0.794	1.21

Sensitivity analysis excluding the participant with the highest hepcidin concentration (1287.5 ng/mL) yielded stable results, with transferrin saturation remaining the sole independent predictor of hemoglobin levels in the multivariable regression model. VIF: Variance Inflation Factor.

Significant values are in bold.

**Fig. 2:**
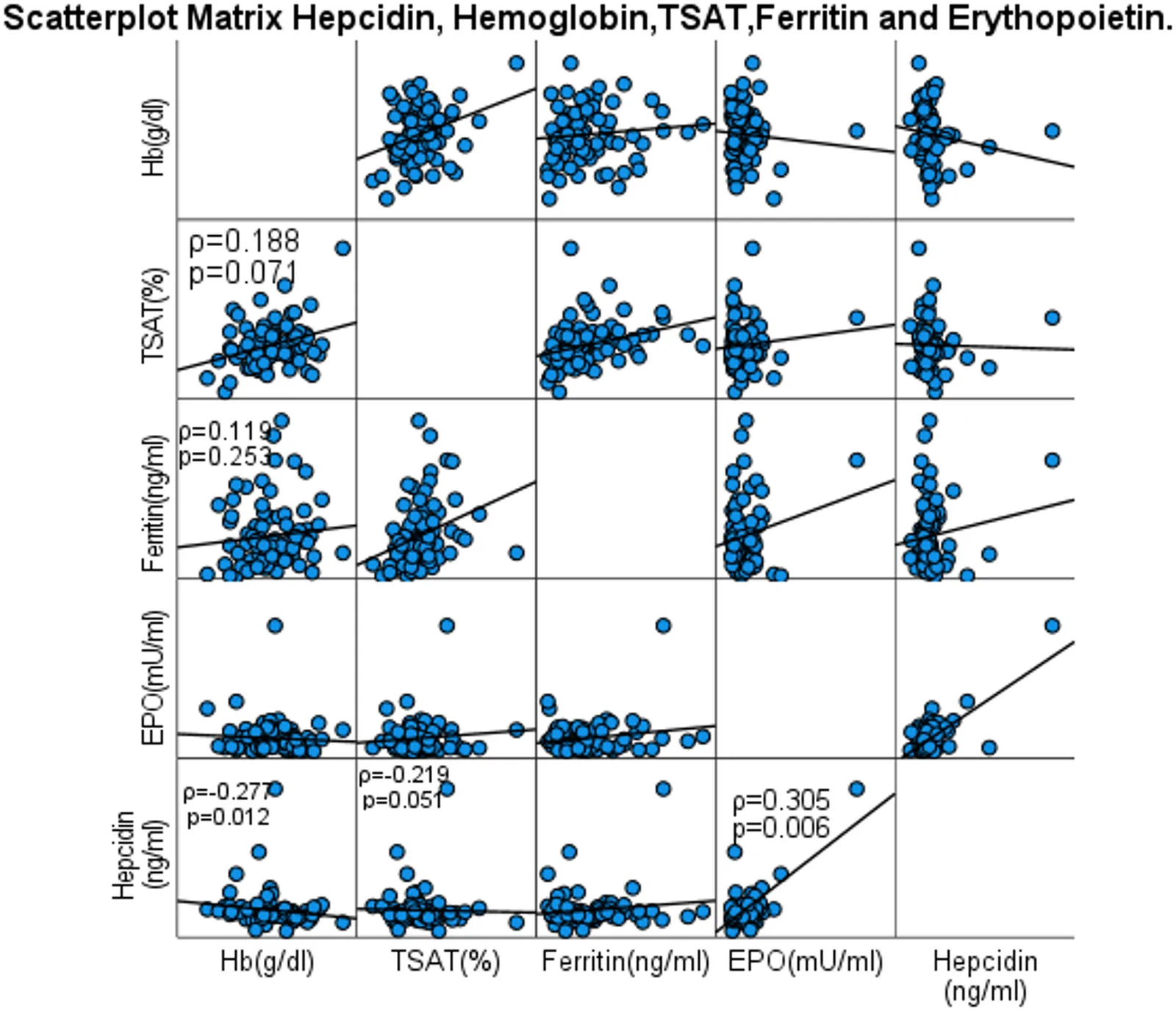
Bivariable correlation matrix of clinical and biochemical parameters. Panels show two-tailed bivariate associations across the sub-cohort (*n* = 81). Unadjusted associations were evaluated using Spearman’s rank correlation coefficient (ρ) for non-parametric data. Sensitivity analysis excluding the extreme hepcidin outlier (1287.5 ng/mL) confirmed that the inverse correlation between hepcidin and hemoglobin remained statistically significant (ρ = -0.287, *p* = 0.010), indicating the bivariate findings were not driven by this single observation.

Figure 2 bivariate analysis showed that serum hepcidin correlated significantly and inversely with hemoglobin (ρ= -0.277, *p* = 0.012) and positively with erythropoietin (ρ = 0.305, *p* = 0.006). However, hepcidin had no significant correlation with transferrin saturation (ρ= -0.219, *p* = 0.051). Hemoglobin levels also showed no significant correlation with baseline transferrin saturation (ρ = 0.188, *p* = 0.071) or ferritin (ρ = 0.119, *p* = 0.252).

Sensitivity analyses excluding the participant with the highest hepcidin concentration (1287.5 ng/mL) yielded similar findings. The inverse correlation between hepcidin and hemoglobin remained significant (ρ= -0.287, *p* = 0.010), and transferrin saturation remained the only independent predictor of hemoglobin in the multivariable regression model. These findings suggest that the primary results were not driven by this extreme observation.

## Discussion

To gain a better understanding of the pathophysiology of CKD-related anemia and to identify potentially helpful interventions, the goal of this study was to examine the intricate interactions among hepcidin, erythropoietin, and anemia in a cohort of nondiabetic CKD patients undergoing follow-up at Mansoura University’s urology and nephrology center.

Our study demonstrated elevated serum hepcidin concentrations in both anemic and non-anemic CKD patients, with no significant difference between the two groups. Although serum hepcidin showed a significant inverse correlation with hemoglobin levels, it was not independently associated with hemoglobin after adjustment for kidney function and iron-related parameters. These findings suggest that the relationship between hepcidin and anemia in CKD is complex and may be influenced by multiple interrelated factors, including renal function and iron metabolism. Hepcidin, a peptide hormone produced primarily by the liver, regulates systemic iron homeostasis through binding to and degradation of the iron export protein ferroportin, thereby reducing iron absorption and mobilization. Because hepcidin is partially cleared by the kidneys, declining renal function may contribute to increased circulating hepcidin concentrations in patients with CKD^16^. Hepcidin at higher than normal levels inhibits iron release from macrophages and enterocytes, leading to decreased iron availability for erythropoiesis^17^. The significant univariable inverse correlation between hepcidin and hemoglobin levels initially supports this relationship; however, its loss of statistical independence in the multivariable model indicates that other factors, particularly transferrin saturation, are more influential in determining hemoglobin status. These findings align with previous studies that have shown variable hepcidin levels in CKD patients and suggest that hepcidin alone may not be a reliable marker for anemia management in this population^18,19^.

While the median endogenous EPO levels in both groups fell within the standard laboratory reference range (~ 4.7 mU/mL), these values are inappropriately low given the degree of anemia present in this cohort. In patients with intact renal function, anemia triggers a robust exponential increase in serum EPO. Therefore, our findings demonstrate a relative EPO deficiency, which is a classic hallmark of anemia of chronic kidney disease, where erythropoietin production fails to scale adequately with declining hemoglobin levels. Erythropoietin, which is produced by the kidneys in response to hypoxia and stimulates red blood cell production in the bone marrow, is impaired by reduced glomerular filtration rates^20^. Decreased erythropoietin levels could not be considered the only factor explaining the different variations in hemoglobin levels in CKD patients. This notion is supported by the lack of significant differences between the study groups. Considering both erythropoietin and other contributing factors, such as iron availability, inflammation, and nutritional status, is crucial to better understand the pathophysiology and management of CKD-related anemia.

In healthy physiological states, an inverse relationship between erythropoietin (EPO) and hepcidin is expected, as EPO-stimulated erythroblasts secrete erythroferrone (ERFE) to downregulate hepatic hepcidin synthesis and mobilize iron for erythropoiesis^21^. However, in our non-dialysis CKD cohort, we observed a significant positive correlation between serum hepcidin and endogenous EPO. This paradoxical co-elevation likely reflects three converging mechanisms inherent to CKD pathophysiology. First, progressive decline in renal function impairs the metabolic clearance of both circulating hepcidin and endogenous EPO, resulting in concurrent systemic accumulation. Second, chronic uremic toxicity and disturbed marrow microenvironments may blunt erythroblast ERFE expression or target-organ sensitivity, disrupting the classic ERFE–hepcidin axis. Third, shared upstream pathophysiological triggers, specifically tissue hypoxia and low-grade systemic inflammation, simultaneously drive compensatory renal/extra-renal EPO secretion and hepatic hepcidin expression. Together, these findings highlight a breakdown of normal homeostatic feedback loops in non-dialysis CKD. Chronically elevated hepcidin concentrations restrict iron recycling and absorption, leading to functional iron deficiency and compromised hemoglobin synthesis. The resulting downstream tissue hypoxia subsequently serves as a continuous physiological stimulus for the renal parenchyma to upregulate endogenous EPO production via hypoxia-inducible factor (HIF) pathways. This parallel movement suggests that despite a relative EPO deficiency relative to the absolute degree of anemia, the fundamental oxygen-sensing mechanism remains functional and closely responsive to hepcidin-mediated iron restrictions in this non-diabetic population. This native compensatory mechanism mirrors the pharmacological axis exploited by modern HIF-prolyl hydroxylase inhibitors such as roxadustat, which intentionally manipulate the HIF-sensing pathway to coordinate reciprocal improvements in hepcidin and hemoglobin parameters even across complex, micro-inflammatory renal cohorts^22^.

Compared with non-anemic patients, anemic patients exhibited lower, although not statistically significant, transferrin saturation (TSAT) levels. Interestingly, a subgroup analysis of our anemic patients (*n* = 20) revealed that only 5 patients (25%) presented with absolute iron deficiency defined by a TSAT < 20%. The remaining 75% of anemic subjects were iron-replete according to TSAT criteria. This crucial distinction highlights that the primary driver of anemia in this non-diabetic CKD cohort is functional iron deficiency, where iron is present in stores but poorly available for erythropoiesis, rather than absolute iron deficiency. In addition to functional iron deficits, absolute iron store depletion remains a significant issue in our cohort. The median ferritin level was approximately 88 ng/mL, and notably, 55 out of 94 patients (58.5%) presented with a serum ferritin below 100 ng/mL. In the context of non-dialysis CKD, current KDIGO guidelines recognize a ferritin level below 100 ng/mL as a key threshold where iron stores are insufficient and a clinical trial of iron supplementation is indicated^11^. This finding carries an important clinical message: despite normal TSAT levels in many subjects, more than half of this non-diabetic CKD population could potentially benefit from targeted iron therapy to optimize hemoglobin production. In addition, Fig. 2 demonstrates a modest positive association between TSAT and hemoglobin levels (*p* = 0.071). Transferrin is the principal iron transport protein in circulation, and TSAT reflects the proportion of transferrin-bound iron available for erythropoiesis^23^. The observed association between TSAT and hemoglobin levels is consistent with the importance of iron availability in maintaining erythropoiesis among patients with CKD. Furthermore, TSAT is independently associated with hemoglobin levels in the multivariable analysis, supporting its potential role as a clinically relevant marker of iron availability in this population^24^.

Figure 2 demonstrates a modest but statistically significant inverse correlation between serum hepcidin and hemoglobin levels. As a key regulator of iron metabolism influenced by inflammation and iron status, elevated hepcidin blocks intestinal iron absorption and macrophage iron recycling, culminating in iron-restricted erythropoiesis and anemia^25^. While the wide range of renal impairment in this cohort (eGFR 25–75 mL/min) likely introduces substantial variability into this relationship, the multivariable regression analysis (Table 5) reveals that serum hepcidin is not an independent predictor of hemoglobin levels when accounting for concurrent clinical covariates. This loss of statistical independence indicates that the univariable association between hepcidin and anemia is largely driven by confounding clinical parameters. These findings align with prior reports in *Nephrology Dialysis Transplantation* demonstrating that systemic inflammatory markers modulate hepcidin independently of hemoglobin^6^. Interestingly, baseline systemic inflammation, as evaluated by the surrogate marker neutrophil-to-lymphocyte ratio (NLR), was comparable between the anemic and non-anemic groups (*p* = 0.444). While this suggests a relatively neutral baseline inflammatory profile, we must acknowledge that direct acute-phase reactants (e.g., CRP or IL-6) were not measured. Therefore, while the independent association of transferrin saturation with hemoglobin appears robust, the possibility of residual confounding by unmeasured micro-inflammatory mediators cannot be entirely excluded. This further supports the notion that hepcidin levels may be more closely related to functional iron stores than to hemoglobin levels in stable CKD populations. Another study reported that hepcidin levels are more closely related to iron stores and inflammation than to hemoglobin levels in CKD patients^26^.

In our stratified analysis by CKD stage, serum levels of erythropoietin and hepcidin did not differ significantly across G2/G3a, G3b, and G4 groups, suggesting that their variability may not be directly stage-dependent within the studied eGFR range. In contrast, evaluation of mineral–bone disorder (CKD-MBD) parameters revealed that intact parathyroid hormone (iPTH) levels were significantly elevated in stage G4 patients, while serum calcium and phosphorus concentrations remained stable and maintained within physiological limits across all stages. This distinct biochemical pattern illustrates early compensatory secondary hyperparathyroidism, wherein PTH secretion progressively rises to preserve normocalcemia and normophosphatemia as GFR declines. Although iPTH was not independently associated with hemoglobin levels in our multivariate model, these findings highlight the early emergence of CKD-MBD disturbances, which may indirectly influence erythropoiesis and bone marrow microenvironments in advanced pre-dialysis CKD.

There was also a weak correlation between hemoglobin and ferritin levels. Ferritin is a marker of iron stores in the body, and its levels can be affected by both iron deficiency and inflammation. Ferritin levels are more closely related to inflammation and iron stores than to hemoglobin levels in CKD patients. These findings suggest that elevated ferritin levels due to inflammation can mask iron deficiency, leading to a weaker correlation with hemoglobin^27^. Another study revealed that in conditions such as CKD, high hepcidin levels can cause functional iron deficiency, where iron is present but not available for red blood cell production. This can result in normal or elevated ferritin levels despite low hemoglobin levels, weakening the correlation^28^.

Importantly, the levels of biochemical markers, such as creatinine, eGFR, iPTH, and lipid profiles, were not significantly different between the anemic and nonanemic groups. This explains why anemia is multifactorial in its pathophysiology in CKD patients. For example, inflammation, oxidative stress, and nutritional deficiencies are known to have significant effects on anemia in CKD patients^7^.

The findings of this study emphasize the importance of iron-targeted therapies in managing CKD-related anemia rather than measuring erythropoietin levels. Improving iron bioavailability has been shown to improve hemoglobin levels and reduce the need for erythropoiesis-stimulating agents (ESAs) in CKD patients^29^. Although only 20 patients were anemic, this subgroup was not used as a binary outcome in regression; instead, hemoglobin was analysed as a continuous variable, which maintains adequate statistical power. The limited impact of hepcidin and erythropoietin on anemia severity suggests that a comprehensive approach addressing multiple pathways is necessary for effective anemia management. This may include the use of new targeted therapies. Hypoxia-inducible factor prolyl hydroxylase inhibitors (HIF-PHIs), a newly emerging therapy that stimulates endogenous erythropoietin production, have improved iron bioavailability^30^.

A major strength of this study is that our cohort explicitly excluded patients with diabetic nephropathy, thereby minimizing the confounding effects of diabetes-related microinflammation that could disturb iron and hormonal homeostasis. Additionally, the entire cohort was completely naïve to prior iron supplementation and erythropoiesis-stimulating agents (ESAs), eliminating treatment-driven fluctuations and ensuring that baseline iron parameters, hepcidin, and erythropoietin measurements reflected treatment-unmodified endogenous states. Furthermore, the exclusion of active systemic infections, by excluding individuals with a fever > 38 °C, positive microbial cultures, or recent antibiotic therapy, helped reduce transient, acute-phase distortions of iron regulatory pathways. Finally, the documentation of a normal baseline neutrophil-to-lymphocyte ratio (NLR) across participants supported a broadly stable baseline inflammatory profile. Together, these design features provided a well-characterized model to evaluate the biological interplay between hepcidin, iron bioavailability, and erythropoiesis in non-diabetic CKD.

Our study has several relevant limitations. First, the study has a relatively small sample size. Additionally, while the missing biomarker data for 13 patients was missing completely at random (due to strict ELISA kit capacities) and therefore did not introduce systematic bias, it restricted our multivariable regression to 80 complete cases, slightly reducing the overall statistical power. Although we measured iPTH, calcium and phosphate, not all patients had complete mineral-metabolism data; thus residual confounding from inadequately controlled secondary hyperparathyroidism may remain. The study was cross-sectional and cannot establish causal relationships. The absence of inflammatory biomarkers may contribute to residual confounding and is a limitation of the study.

In addition, the study was powered to detect moderate correlations (approximately *r* = 0.30). While a statistically significant inverse correlation between hepcidin and hemoglobin was identified, the observed effect size was modest (ρ= −0.277). Consequently, the precision of this estimate may be limited, and larger studies are warranted to confirm the robustness and clinical relevance of this association.

An important limitation of this study is the absence of direct acute-phase inflammatory biomarkers such as C-reactive protein (CRP), high-sensitivity CRP, or interleukin-6. Although we successfully utilized the neutrophil-to-lymphocyte ratio (NLR) as a validated surrogate marker to confirm the absence of major systemic inflammation, the specific cytokine-driven inflammation–hepcidin–anemia pathways could not be directly quantified. Therefore, residual confounding by unmeasured micro-inflammatory mediators cannot be entirely excluded.

Third, our strict selection criteria and geographic setting impose limits on the external generalizability of our findings. By excluding patients with diabetes mellitus, we eliminated a major real-world CKD subpopulation (representing 30–40% of standard cohorts). While this exclusion was necessary to isolate iron and hepcidin dynamics from the profound confounding influence of diabetic inflammation, it means our results may not apply to diabetic nephropathy. Additionally, this study was conducted at a single tertiary center in Egypt, where distinct regional dietary patterns and endemic infections may uniquely influence iron kinetics. For instance, our study did not screen for Helicobacter pylori infection, which is highly prevalent in the Egyptian population and classically causes iron deficiency via impaired gastric absorption and chronic micro-bleeding, nor did we evaluate historical or active Schistosomiasis exposure, which can drive chronic immune-mediated blood loss. These unmeasured regional factors represent an important caveat when extrapolating our absolute iron and hepcidin profiles to Western or multi-ethnic CKD populations.

Finally, for the purpose of descriptive grouping, we utilized a uniform hemoglobin cutoff to define anemia rather than the sex-specific criteria recommended by the WHO and KDIGO guidelines (Hb < 13.0 g/dL for men and < 12.0 g/dL for women)^11^. While this uniform approach simplified initial comparisons, it may have introduced minor misclassification in our descriptive statistics, potentially underdiagnosing mild anemia in men or overestimating its prevalence in women. However, it is crucial to emphasize that this definition did not influence our main statistical findings, as our primary multivariable regression models analyzed hemoglobin strictly as a continuous variable, ensuring that the relationships between iron parameters, hepcidin, and erythropoiesis remained robust and unaffected by categorical cutoffs.

## Conclusion

In conclusion, this study demonstrates that transferrin saturation (TSAT), reflecting functional iron bioavailability, is the primary independent correlate of hemoglobin levels in non-diabetic chronic kidney disease. Although hepcidin is a recognized key regulator of iron metabolism, neither hepcidin nor erythropoietin maintained an independent association with hemoglobin after multivariable adjustment. Furthermore, utilizing the neutrophil-to-lymphocyte ratio (NLR) suggested a relatively neutral baseline inflammatory profile across the cohort. Although direct inflammatory biomarkers are lacking, these NLR findings imply that anemia in this population may be primarily driven by functional iron handling deficits rather than overt systemic inflammation. Ultimately, these findings emphasize that evaluating and optimizing functional iron availability remains the most critical clinical target for managing anemia in stable, non-diabetic CKD patients.

## Supplementary Information

Below is the link to the electronic supplementary material.

Supplementary Material 1

Supplementary Material 2

## Data Availability

The datasets used and/or analysed during the current study are available from the corresponding author on reasonable request.

## Abbreviations

ALP: Alkaline Phosphatase
BMI: Body Mass Index
CBC: Complete Blood Count
CKD: Chronic Kidney Disease
Cr: Creatinine
EPO: Erythropoietin
eGFR: Estimated Glomerular Filtration Rate
ELISA: Enzyme-Linked Immunosorbent Assay
ESAs: Erythropoiesis-Stimulating Agents
Hct: Hematocrit
HDL-C: High-Density Lipoprotein
HIF-PHIs: Hypoxia-Inducible Factor Prolyl Hydroxylase Inhibitors
Hb: Hemoglobin
HTN: Hypertension
iPTH: Intact Parathyroid Hormone
LDL-C: Low-Density Lipoprotein
MCH: Mean Corpuscular Hemoglobin
MCV: Mean Corpuscular Volume
Mg: Magnesium
NLR: Neutrophil to lymphocyte ratio
PCK: Polycystic Kidney Disease
TIBC: Total Iron-Binding Capacity
TG: Triglycerides
TSAT: Transferrin Saturation
UA: Uric Acid
VIF: Variance Inflation Factor
